# Acute Sleep Restriction Has Differential Effects on Components of Attention

**DOI:** 10.3389/fpsyt.2018.00499

**Published:** 2018-10-30

**Authors:** Jasmyn E. A. Cunningham, Stephanie A. H. Jones, Gail A. Eskes, Benjamin Rusak

**Affiliations:** ^1^Institute of Medical Science, University of Toronto, Toronto, ON, Canada; ^2^Department of Psychology and Neuroscience, Dalhousie University, Halifax, NS, Canada; ^3^Department of Psychology, Acadia University, Wolfville, NS, Canada; ^4^Department of Psychiatry, Dalhousie University, Halifax, NS, Canada; ^5^Chronobiology and Sleep Program, Nova Scotia Health Authority, Halifax, NS, Canada

**Keywords:** vigilance, orienting, executive function, sleep restriction, DalCAB, attention, adult, sleep loss

## Abstract

Inadequate nightly sleep duration can impair daytime functioning, including interfering with attentional and other cognitive processes. Current models posit that attention is a complex function regulated by several separate, but interacting, neural systems responsible for vigilance, orienting, and executive control. However, it is not clear to what extent each of these underlying component processes is affected by sleep loss. The purpose of this study was to evaluate the effects of acute sleep restriction on these attentional components using the Dalhousie Computerized Attention Battery (DalCAB). DalCAB tasks were administered to healthy women (aged 19–25 years) on two consecutive mornings: once after a night with 9 h time in bed (TIB), and once again after either another night with 9 h TIB (control condition, *n* = 19) or after a night with 3 h TIB (sleep restriction condition, *n* = 20). Self-ratings of sleepiness and mood were also obtained following each sleep condition. Participants showed increases in self-reported sleepiness and fatigue after the second night only in the sleep restriction group. Sleep restriction primarily affected processing speed on tasks measuring vigilance; however, performance deficits were also observed on some measures of executive function (e.g., go/no-go task, flanker task, working memory). Tasks assessing orienting of attention were largely unaffected. These results indicate that acute sleep restriction has differential effects on distinct components of attention, which should be considered in modeling the impacts of sleep loss on the underlying attentional networks.

## Introduction

Most adults need, on average, about 8 h of sleep per night, and sleeping <6 h nightly is associated with decreased daytime functioning, poorer general health, increased risk of cardiovascular and metabolic diseases and an increased likelihood of accidents (1–5). However, many people do not get enough sleep daily because of conflicting obligations, personal choices, health conditions, and other factors. Both acute and chronic sleep restriction are especially common among college-aged students [e.g. (6)].

Attention is a fundamental mechanism underlying cognitive abilities that is affected by sleep debt. Experimentally shortened sleep impairs attention in children (7, 8), and impaired attention related to reduced sleep in members of the military causes performance deficits in reaction times and accuracy, as measured by the Attention Network Test (ANT), and in continuous visual tracking (9). Thus, the impacts of sleep loss on attention, and the consequences for cognitive performance, are far-reaching and may be applicable to a wide range of both clinical and otherwise healthy populations.

The model of attention proposed by (10) includes three functional systems that depend on separate, but interacting neural networks: vigilance/alerting; orienting/selection; and executive control (or regulation of attentional resources). The relative independence of these systems is substantiated by evidence that performance in these domains can differ significantly within individuals (as assessed using tests such as the ANT), and that there are low correlations among network scores (11, 12).

The proposed vigilance and alerting network is involved in preparing and maintaining attention to attend to high-priority stimuli and signals, and is strongly associated with the brain's norepinephrine neurotransmitter system. There are two types of alertness: tonic, which involves lengthy and sustained vigilance, and phasic, which involves shifting into an alert state in response to an acute internal or external event. Alertness does not affect the rate of information accumulation, which underlies accuracy in choice situations, but it affects response speed. Thus, increased alertness is associated with faster responses to stimuli, but at the risk of a higher rate of errors, as participants choose a response more quickly but with potentially less information upon which to base their decisions (4, 10, 12).

The second proposed network, orienting, relates to searching and selecting stimuli for further processing. In the original model, one orienting network involving parietal, frontal, and subcortical areas of the brain was proposed to be responsible for prioritizing and orienting to sensory events by location and modality (10). More recently, two (sub)networks have been proposed to be involved in these processes (12). For the purposes of this study, we will refer to a single orienting network, given the prevalence of this model in the relevant literature and its incorporation into many attention tasks, and the fact that the visual search tasks used in this study incorporate aspects of both orienting subnetworks.

The third proposed network is executive control, which is responsible for allocating attentional resources. While originally one executive control network was proposed to be responsible for detecting signals from all sources (10), more recently, (12) proposed two separate subnetworks of executive control that stem from a common developmental origin. While cognitive theories differ with respect to whether executive control operates as a single system or not, the functions served by executive control are very similar in both views (12).

A variety of different measurement tools, including the Psychomotor Vigilance Task (PVT), the ANT, the Sternberg item memory test and go/no-go tests have been used in the past to measure the impact of sleep loss on attentional performance. Various amounts of sleep loss have also been studied, including total sleep deprivation (remaining awake for one or more full nights); acute partial sleep restriction (receiving less than habitual amounts of sleep for one night); and chronic partial sleep restriction (receiving less than habitual amounts of sleep over a period of several days). See (13) for a review. Despite the large amount of previous research, there remain gaps in our understanding of how sleep loss affects different functional aspects of attention.

It is widely accepted that vigilance is reliably lowered by total sleep deprivation (2, 3, 14–16) and chronic (3, 14) and acute (2) sleep restriction, although there have been fewer studies on the effects of acute partial sleep loss. It is less well understood how sleep loss affects orienting and executive functions, and studies examining broader aspects of attention (e.g., using the ANT) have produced inconsistent results [e.g., (4, 9, 17)]. The reasons for this lack of clarity likely include the fact that the performance of separate attentional networks is often compared between, rather than within, studies and the impact of the “task impurity” problem. Task impurity refers to the fact that many tasks used to measure executive functioning assess a complex combination of executive skills, including working memory, inhibition and task switching. It is therefore difficult to assign performance declines after sleep loss to effects on one or more individual component processes (13). Given that tests across studies also differ in task demands, it is difficult to determine whether these attentional processes are differentially sensitive to sleep loss.

The purpose of this study was to investigate the differential impact of acute, partial sleep restriction on all three attentional networks. Given the limitations of available measures of attention, the Dalhousie Computerized Attention Battery (DalCAB) was developed, based on the Posner and Peterson model (10), to simultaneously assess the vigilance, orienting, and executive control networks of attention. The DalCAB includes eight psychometrically stable computerized tasks that assess these networks, and has been shown to have good test-retest reliability (18) as well as construct validity as a measure of all three networks (19). In the present study, healthy female participants were assigned to either sleep restriction or control groups. For both groups, the DalCAB was administered on two separate occasions, once after a 9 h sleep opportunity, and again after either a 3 h sleep opportunity or after another 9 h sleep opportunity (to control for practice effects).

Based on consistent findings in the literature related to the effects of sleep loss on vigilance (2–4, 14, 15), we predicted that vigilance performance would be significantly impaired following sleep restriction after controlling for possible practice effects. The less consistent literature related to effects of sleep loss on orienting and executive control (4, 9, 16, 17) did not allow a prediction as to how the function of these networks would be affected.

## Methods

### Participants

Because resource limitations did not allow studying an adequate number of participants of both sexes, and women are less frequently studied, and often without adequate consideration of potential effects of menstrual cycle phase, only female participants were included. Sleep characteristics and physiological responses to sleep loss have been shown to be affected by menstrual cycle phase (20, 21); thus, all participants were studied during the mid-follicular phase of their menstrual cycle, as determined by the self-reported timing of two immediately preceding menstrual cycles.

Thirty-nine healthy women (aged 19–25 years, mean age 21 years) were recruited from the community in Halifax, Nova Scotia, Canada. Inclusion criteria were: a self-reported habitual sleep duration of 7–9 h, confirmed by sleep diaries 7 days prior to study participation; no regular daytime naps; no history of sleep disorders; no intake of caffeinated beverages exceeding the equivalent of two cups of coffee (~300 mg caffeine) daily; no recent history of shift work or recent (past 6 weeks) transmeridian flights crossing more than two time zones; no history of regular recreational drug use; no history of neurological disorders or current psychiatric disorders; non-extreme chronotype, as determined by a Morningness-Eveningness Questionnaire [MEQ; (22)]; no diagnosis of head injury with a loss of consciousness in the past 5 years; currently taking oral contraceptives regularly; normal or corrected-to-normal vision and hearing; body mass index between 18.5 and 25.0; and no experience of general anesthesia during the last 4 months.

### Tests

The DalCAB comprises eight reaction time (RT) tasks (Table 1), each designed to measure the processes underlying different components of attention by collecting RTs and accuracy. These include: vigilance, orienting, and executive control components (19). The DalCAB tasks are as follows:

**Table 1:** Dalhousie Computerized Attention Battery (DalCAB) tasks, task functions and task-specific variables.

**Proposed network**	**Task^*a*^**	**Function**	**Task-specific variables**
Vigilance	Simple RT	Response speed	Response Stimulus Interval (RSI)
	Choice RT	Decision response speed	Response switch
	Feature visual search	Search and select	Distractor set size
Orienting/selection	Conjunction visual search	Search and select	Distractor set size
Executive	Go/no-go	Inhibition	Go frequency
	Dual task	Dual task	Response switch
	Flanker	Filtering, response conflict	Congruency of flankers
	Item memory	Verbal working memory	Set size, trial type
	Location memory	Spatial working memory	Set size, trial type

a *Network designations determined by factor analysis in (19). Tasks are sorted into the different attentional networks proposed by (10)*.

#### Simple reaction time (SRT; vigilance)

The reaction time from stimulus onset to button press was used to measure response speed. Response-stimulus intervals (i.e., the length of time between participant response and subsequent stimulus presentation) varied between 500 and 1500 ms.

#### Choice reaction time (CRT; vigilance)

A two-choice reaction time task measured decision response speed. Response switching effects were also calculated on consecutive trials that required a different response choice as compared to consecutive trials requiring the same response.

#### Visual search (orienting; vigilance)

Participants searched and made an orientation judgment to colored stimuli among different set sizes of distractors ranging from 6-18 items. Targets were defined by a single feature (e.g., color, termed feature search) or by the combination of 2 features (e.g., color and shape; termed conjunction search).

#### Dual task (executive)

Control of attentional resources was measured by combining the choice reaction time (CRT, described above) task with a counting task that involved responding to each trial as above, while simultaneously keeping track of how many times each of the 2 stimulus choices were presented.

#### Flanker task (executive)

A flanker task assessed response conflict resolution and filtering (an aspect of executive control). Choice reaction time to identify the shape of a central stimulus with the same (congruent) or different (incongruent) vertical flanking stimulus shapes was measured. The congruency effect was calculated by subtracting the RTs or accuracy in the congruent condition from those in the incongruent condition.

#### Go/No-Go (GNG) task (executive)

The go/no-go task was used to measure initiation and inhibition. Participants were required to respond to certain stimuli (“go”), and to inhibit responses to others (“no-go”). The frequency with which “go” stimuli appeared varied from 20 to 80% across blocks of trials.

#### Item working memory task (executive)

The item working memory task presented a series of non-repeating stimuli (2–6 items), followed by a probe target stimulus after a delay. Participants were required to identify whether the target stimulus was present in the preceding series of stimuli.

#### Location working memory task (executive)

The location working memory task presented a stimulus at different sequential locations (2–6 locations), followed by a probe target stimulus. Participants were required to identify whether the target stimulus location was included or not included in the previous sequence.

### Mood and sleepiness scales

#### Morningness-eveningness questionnaire

All participants completed the Morningness-Eveningness Questionnaire (22) prior to participation in the study to ensure that they were not extreme morning or evening types (usually referred to as “chronotypes”). The questionnaire included 13 questions, each with four or five answer options, and was scored such that a higher score on a question indicated a morning chronotype, and a lower score indicated a more evening chronotype. The possible range of scores was from 13 to 55; a score between 23 and 43 was required for inclusion, as scores outside this range indicate extreme morning or evening chronotypes (22).

#### Profile of mood states (POMS)

Participants completed the Profile of Mood States (23) each evening and morning of the study, in order to assess self-reported changes in Tension-Anxiety, Depression-Dejection, Anger-Hostility, Vigor-Activity, Fatigue-Inertia, and Confusion-Bewilderment in conjunction with sleep restriction. Each of these six mood factors was scored individually, with a higher score indicating a stronger mood state. Factors were then summed (or subtracted, in the case of the Vigor-Activity score) to produce a Total Mood Disturbance Score. In the case of missing items, the mean of all completed items contributing to the same factor was used as an imputed item score (23).

#### Stanford sleepiness scale (SSS)

Participants completed the Stanford Sleepiness Scale each morning of the study (24, 25) to quantify changes in subjective sleepiness. The scale consists of seven ranked statements, with higher scores indicating more sleepiness. Participants were asked to record the rank of the statement that most accurately represented their current degree of sleepiness.

### Apparatus

DalCAB stimuli were presented on a 17″ monitor of an Apple iMac G5 computer. Participant responses were collected using a Razer DeathAdder Gaming Mouse (sensitive to 1 ms response times), using the left and right mouse buttons.

### Procedure

Individuals interested in participating were first screened with respect to preliminary inclusion criteria during a telephone call. Eligible participants completed a screening questionnaire and the MEQ to confirm eligibility. After written informed consent was obtained, participants were assigned to either the control (C; *n* = 19) or sleep restriction (SR; *n* = 20) group. The first ten SR participants were recruited prior to the addition of C participants, after which group assignment was randomized. Each participant was scheduled to participate in the laboratory portion of the study near the midpoint of the follicular phase of her menstrual cycle. Participants completed a sleep diary every morning during the week prior to this point.

**Figure 1:**
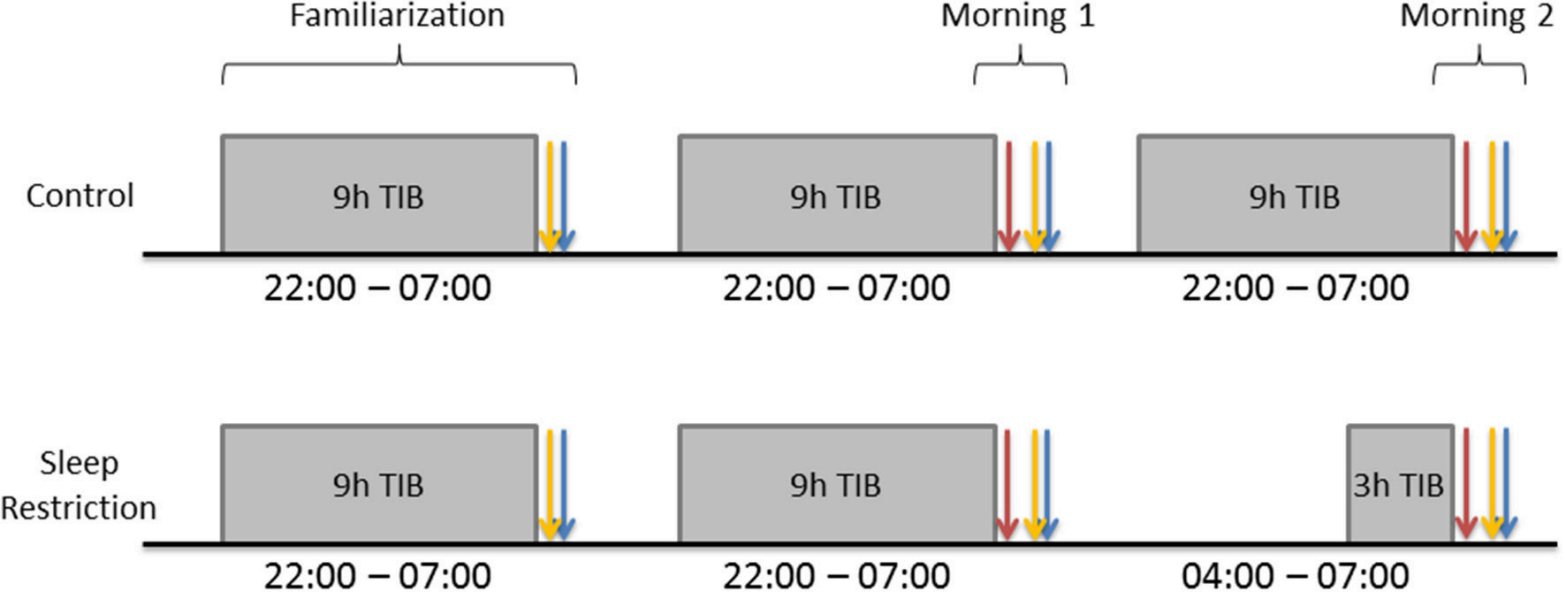
Gray rectangles represent participant sleep opportunities. Blue arrows represent Profile of Mood States (POMS) completion. Yellow arrows represent Stanford Sleepiness Scale (SSS) completion. Red arrows represent Dalhousie Computerized Attention Battery (DalCAB) completion. DalCAB was always started within 1 h of waking in the mornings.

Participants spent three consecutive nights (Figure 1) in the Chronobiology Laboratory at the QE II Health Sciences Centre (Halifax, Nova Scotia). The first night served to adapt participants to sleeping in the laboratory. They arrived at 21:30, completed the Profile of Mood States (POMS) and were allowed to sleep in a darkened bedroom from 22:00 to 07:00. The following morning, they completed the POMS and Stanford Sleepiness Scale (SSS) within an hour of awakening, were provided with breakfast (without a caffeinated beverage), and were instructed to go about their normal daily schedule without daytime napping or caffeine intake. The same procedures were followed on the second night. The following morning (Day 1), participants completed the POMS, SSS and started the DalCAB within an hour of waking. After completing the DalCAB (~1 h), participants were provided with breakfast (without a caffeinated beverage) and resumed their normal daily schedule, without daytime naps or caffeine intake.

During the third night, procedures for C participants were identical to those on the preceding night, with 9 h time in bed (TIB; 22:00–07:00). SR participants also arrived by 21:30 on the third night and completed the POMS. They then stayed awake until 04:00 and were allowed 3 h TIB until they were awakened at 07:00. The following morning (Day 2), both C and SR participants completed the POMS, SSS, and DalCAB, as on Day 1. During the time that SR participants stayed awake overnight, they participated in non-strenuous activities, such as reading, playing video or board games, listening to music, watching videos, or working on a computer, while continuously in the company of a research assistant. Participants in the SR group were not permitted to drive after sleep restriction, so they were sent home either by an arranged ride or taxi, if transportation was needed.

Participants received CAD $125 as compensation for their time and effort in participating in the study. The study protocol was approved by the Capital District (now Nova Scotia) Health Authority Research Ethics Board, in conformity with the Canadian Tri-Council Policy Statement 2: Ethical Conduct of Research involving Humans (2014).

### Data analysis

All statistical analyses were performed in R version 3.1.1 (R, Retrieved from http://www.r-project.org/). *P* values < 0.05 were considered statistically significant. Participant demographics were compared between groups using unpaired *t*-tests, with the exception of handedness which was compared using a chi squared test for independence. POMS and SSS scores in the morning were compared across days and treatment groups, using mixed design analysis of variance (ANOVA). POMS scores were assessed as individual mood dimensions.

Reaction times (RTs), measured in ms, were collected for each DalCAB task. Only correct responses >100 ms which fell within the trial length [varied by task, see (18)] were included in analyses of RTs for all tasks. Accuracy was also calculated for each task, with accuracy defined specifically for each task (as described in Results). Anticipations were defined as RTs ≤ 100 ms. RTs, accuracy (% correct), and anticipations (as applicable) were compared across mornings (Day 1 vs. Day 2) and between groups (C vs. SR), as well as across the different levels of the task-specific variables, using mixed design ANOVA. Statistically significant results from the ANOVAs were followed by *post-hoc* tests for both accuracy and RTs using Tukey's HSD for multiple comparison correction. With the exception of demographic data, only significant results are reported in the text. All findings and details of the RT ANOVA results are included in the Supplementary Material.

## Results

### Demographics

Control and sleep restriction groups did not differ significantly with respect to age, years of education, body mass index, handedness, MEQ score or baseline sleep diary total sleep time; see Table 2.

**Table 2:** Participant demographic information.

**Attribute**	**Control group**	**Sleep restriction group**	***p*-value**
*n*	19	20	–
Age (years)	20.7 ± 0.38	21.3 ± 0.42	0.33
Education (years)	14.5 ± 0.39	15.3 ± 0.39	0.18
BMI	22.5 ± 0.51	22.7 ± 0.44	0.70
Handedness (Right)	84%	95%	0.56
MEQ score	34.4 ± 1.56	35.5 ± 0.94	0.58
Sleep diary TST (avg. hours)	7.8 ± 0.16	7.9 ± 0.18	0.58
Sleep diary sleep efficiency^*a*^ (avg. %)	90.6 ± 0.97	91.2 ± 1.02	0.68

a *Proportion of time in bed spent asleep. Values shown are the mean ± SEM*.

### Mood and sleepiness scores

**Figure 2:**
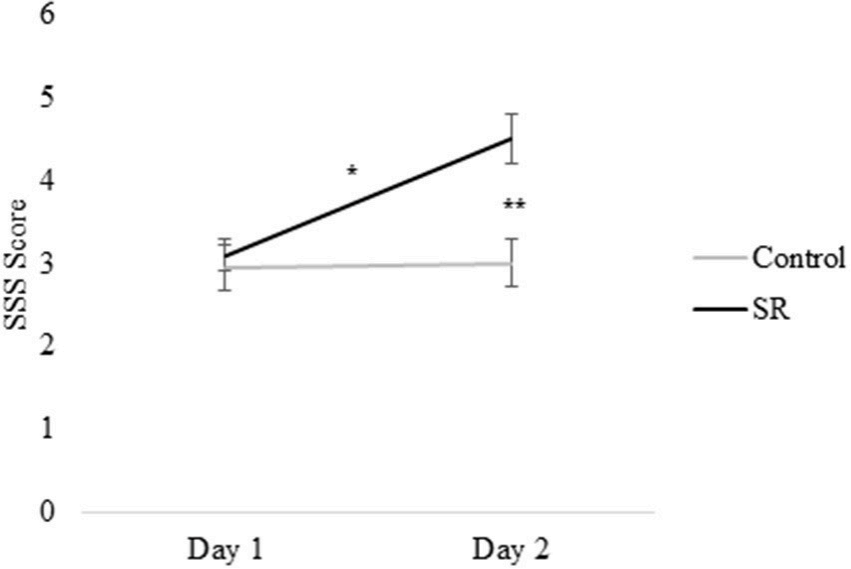
Stanford Sleepiness Scale (SSS) scores on Day 1 and Day 2. In all figures, error bars indicate the standard error of the mean. In all figures, ^#^*p* < 0.05; ^*^p < 0.01; ^**^p < 0.001.

**Figure 3:**
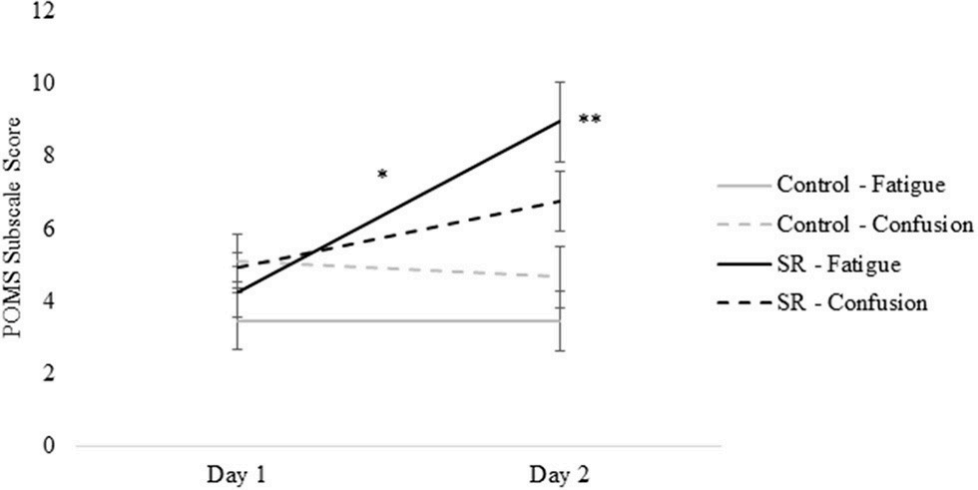
Profile of Mood States (POMS) subscale scores for Fatigue and Confusion on Day 1 and Day 2.

Figures 2, 3 present self-reported sleepiness (SSS) and fatigue and confusion scores (subscales of the POMS), respectively, across days for both C and SR groups. An interaction between day and group for SSS scores (*p* = 0.001; Figure 2) revealed a significant change in sleepiness from Day 1 to Day 2 for SR participants (*p* = 0.002), and a significant difference between C and SR participants on Day 2 (*p* < 0.001). Similarly, significant interactions between day and group were found for fatigue (*p* < 0.001) and confusion (*p* = 0.02) (Figure 3). Fatigue scores increased significantly from Day 1 to Day 2 for the SR group (*p* = 0.001) and differed between the C and SR groups on Day 2 (*p* < 0.001). No *post-hoc* tests of POMS confusion scores were significant.

### DalCAB performance

For each DalCAB task, only significant Group X Day interactions for accuracy and reaction time (RT) outcomes are reported (see Supplementary Material for all RT ANOVA results).

### Vigilance performance (simple and choice reaction time, feature visual search)

**Figure 4:**
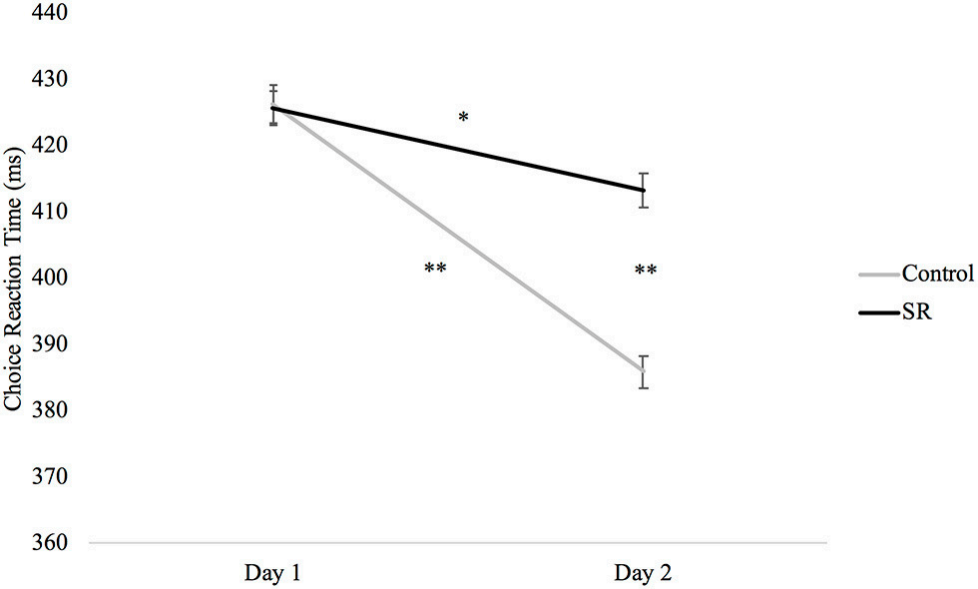
Reaction times (ms) by Day and Group for the Choice Reaction Time task.

Figure 4 plots RTs (in ms) in the Choice Reaction Time task across days and groups. A significant interaction between group and day (*p* = 0.02) revealed that RTs in both groups were faster on Day 2 than Day 1 (SR: *p* = 0.004; C: *p* < 0.001), but that the SR group was significantly slower than the C group on Day 2 (*p* < 0.001).

The group by day interaction was also significant for anticipations on the Simple Reaction Time Task (*p* = 0.02), but no further comparisons were significant (Tukey's *post-hoc* tests).

**Figure 5:**
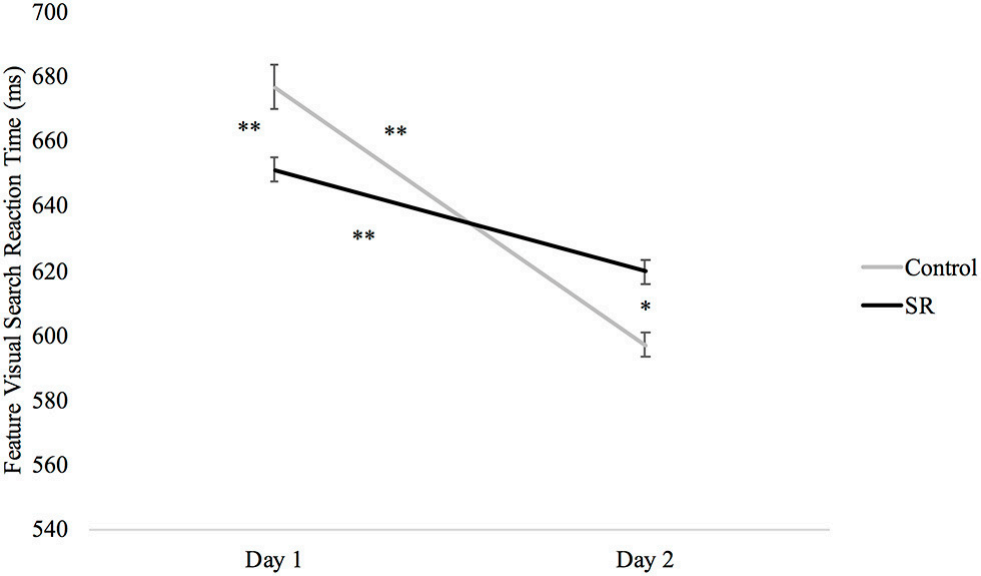
Reaction times (ms) by Day and Group for the Feature Visual Search task.

Figure 5 plots RTs in the Feature Visual Search task across days and groups. While RTs were significantly faster for both groups on Day 2 than on Day 1 (SR: *p* < 0.001; C: *p* < 0.001), RTs for the SR group were faster on Day 1 (*p* < 0.001), but slower on Day 2 (*p* = 0.003), when compared to the C group.

### Executive performance (Go/No-Go, item memory, vertical flanker)

**Figure 6:**
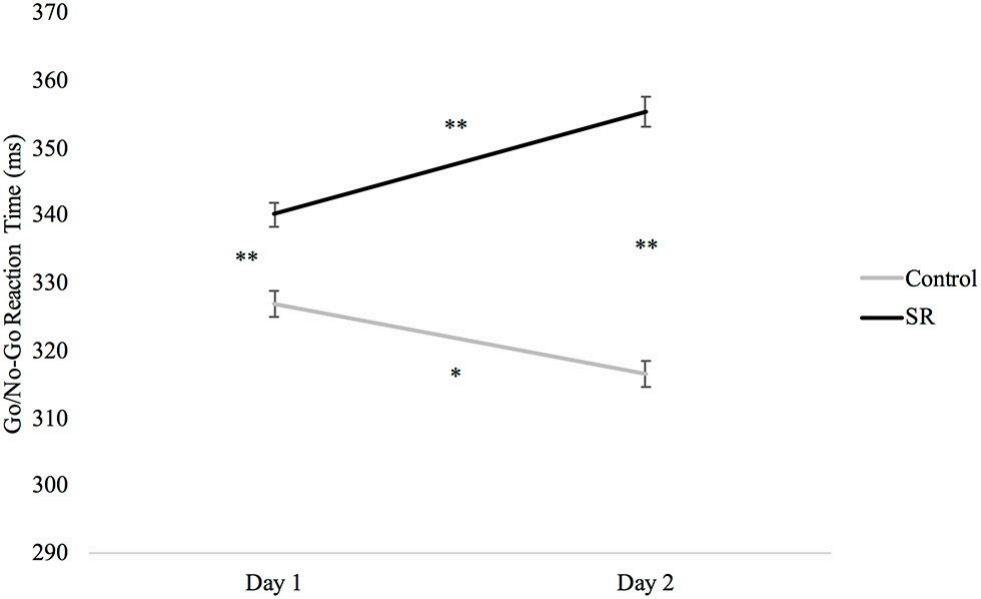
Reaction times (ms) by Day and Group for the Go/No-Go task.

Analyses of the Go/No-Go task revealed slower RTs after sleep restriction (*p* < 0.001; Figure 6). Participants in the C group showed significantly faster RTs on both days when compared to the SR group (Day 1: *p* < 0.001; Day 2: *p* < 0.001). The SR group, however, showed slower RTs on Day 2 than on Day 1 (*p* < 0.001), while the C group showed faster responses on Day 2 than on Day 1 (*p* = 0.001).

**Figure 7:**
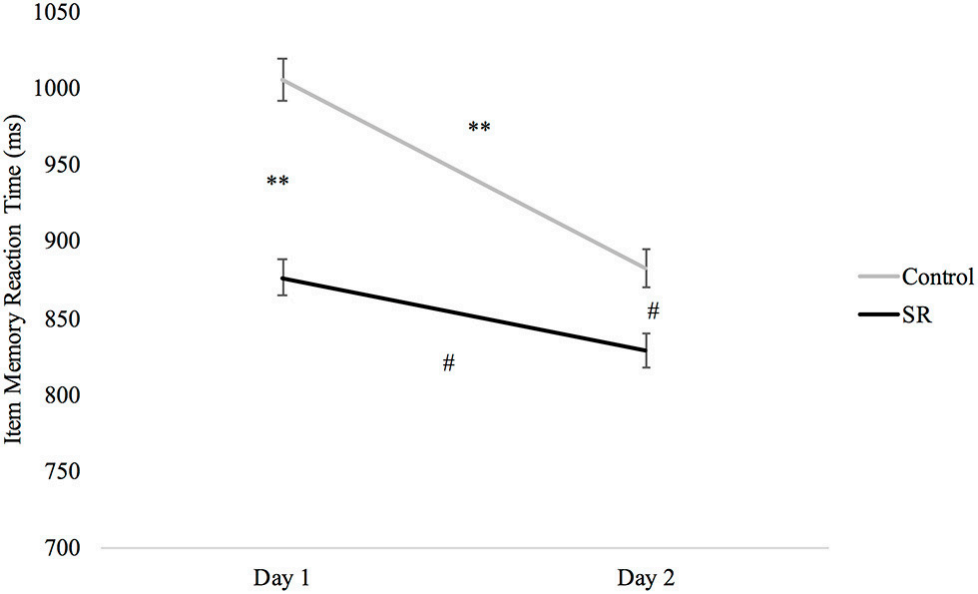
Reaction times (ms) by Day and Group for the Item Memory task.

A significant Group X Day interaction on the Item Memory task (*p* = 0.039) revealed significantly faster RTs in the SR group than the C group on both Day 1 (*p* < 0.001) and Day 2 (*p* = 0.012), and faster RTs in both groups on Day 2 (C: *p* < 0.001; SR: *p* = 0.033). There was, however, less improvement in the SR than in the C group between Days (Figure 7).

**Figure 8:**
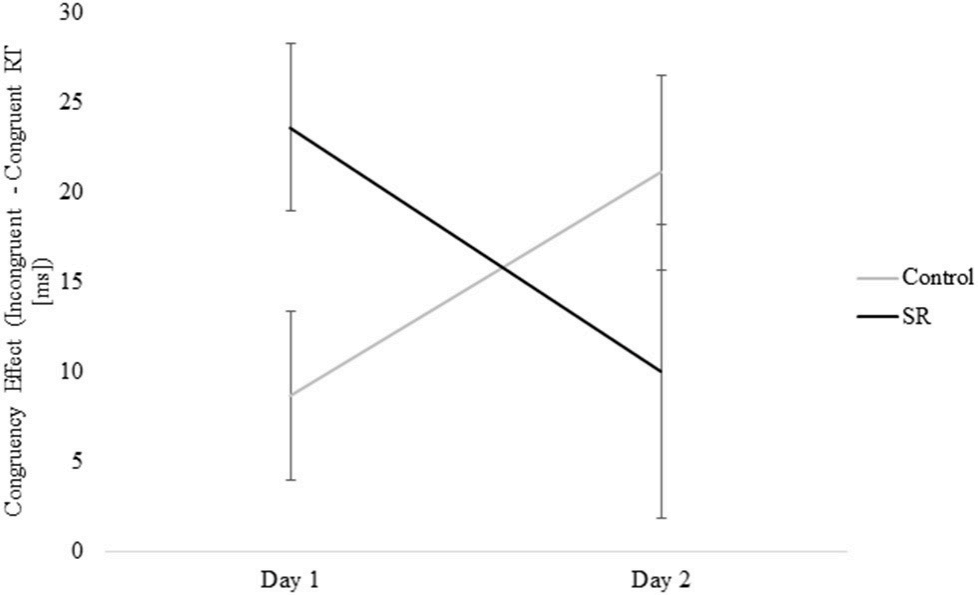
Measure of the Congruency Effect (Incongruent – Congruent RTs [ms]) by Group and Day for the Vertical Flanker Task.

Figure 8 shows the significant interaction between Day and Group on Flanker Congruency effects on the Vertical Flanker task (*p* = 0.03). No further comparisons were significant (Tukey's *post-hoc* tests).

## Discussion

This study was conducted to investigate the impact of acute, partial sleep restriction simultaneously on all three theoretical attentional networks: vigilance, orienting, and executive control (10). The DalCAB allows for a direct comparison of performance changes related to sleep loss on the three networks using a common methodology during a single 1 h test session.

The SSS scores and POMS Fatigue and Confusion scores on Day 2 showed that restriction for a single night to 3 h TIB effectively increased subjective sleepiness and associated mood changes, as expected (2, 14), while those with 9 h TIB showed no significant changes in these measures on Day 2.

The vigilance network, responsible for preparing and maintaining readiness to respond (10), was primarily measured in the DalCAB by the Simple and Choice RT tasks. For the Choice RT task, while both groups showed significant practice improvements on Day 2, there was less improvement in the SR group and a significant RT difference between the two groups on Day 2 that was not present on Day 1. These results are consistent with the known effects of sleep restriction in reducing vigilance (4, 26, 27), slowing reaction times (3, 4, 26–29), and increasing lapses and other errors (2, 4, 14, 15, 30) on RT tasks.

A factor analysis of DalCAB variables showed that mean RTs from the Feature Visual Search task also assessed the function of the vigilance network of attention (19). SR participants showed significantly slower RTs on this task than the C group on Day 2, even though they initially had faster RTs on Day 1. Thus, the results on the tasks that measured vigilance indicate that SR reduced the function of this network as expected.

The literature related to sleep loss effects on the orienting network of attention is inconsistent, in part because of the use of different methodologies and study populations (4, 7, 9, 17, 28). In this study, the orienting component of attention was assessed with the Conjunction Visual Search task. There was no significant group by session interaction on outcomes of this task, in either reaction time or accuracy data, thereby indicating no effect of sleep loss on orienting performance, which is consistent with some (4, 9, 17) but not other (27) previous findings.

One possible contributor to the inconsistency in the published literature is variation in what is being measured using different orienting tasks. Various orienting tasks use different paradigms to assess the deployment of spatial attention, often by using a combination of invalid, valid, and neutral visual cues preceding a target stimulus. In the DalCAB Conjunction Visual Search task, participants were not cued to their target stimulus, and relied on internal control. This method is comparable to the neutral cues often used in control trials in similar studies. In these cases, reaction times to neutral cues are often the same before and after sleep loss (26, 31), even when there is impairment due to sleep loss on RTs for invalid cues (31). Thus, it is possible that internal control of spatial orienting is less affected by sleep loss than is external control (i.e., by cueing) of spatial attention.

This result may be specific to the costs of invalid cues; some studies have found that RTs on validly cued trials are not affected by sleep loss (26, 31). Additionally, orienting to a new sensory stimulus has been modeled as consisting of three component steps: disengaging attention from a previous target; looking (moving the gaze); and seeing (discriminating a new target) (26, 31–33). It has been suggested that it is the disengagement step, required for invalid cue conditions, which is most impacted by sleep loss (32). On the other hand, another study found that the RT costs of invalid cues actually go down after sleep loss (4). Alternatively, the degree of sleep loss in this study may not have been sufficient to impair the orienting network to a degree that could be measured by the conjunction visual search task.

Executive control of attention comprises several different processes that have been assessed using a variety of tasks. Some studies have reported impairment of executive function after sleep loss (9, 17, 27, 29, 34) while others have not (4, 7, 28, 34, 35). This variability probably reflects the use of different tasks and the fact that some tasks may target different aspects of executive control and/or the use of tasks with multiple components that do not target executive function specifically. Three of the five tasks that tap into executive mechanisms in this study showed impairment after sleep loss (i.e., Go/No-Go, Flanker, and Item Memory tasks). On the Go/No-Go task, C participants showed faster responses on Day 2, while SR participants showed slower responses. On the Flanker task, only the C group improved from Day 1 to Day 2. In addition, the performance benefit of stimulus congruity was lost on Day 2 for the SR group, but not the C group. Similarly, performance on the Item Memory task improved for both groups on Day 2, but the improvement was significantly greater for the C group. In contrast to these findings, sleep restriction did not impair performance on the Location Memory or Dual tasks.

Based on these results, we can conclude that some components of executive function—inhibition (Go/No-Go), filtering (Flanker), and verbal working memory (Item Memory), were impaired by sleep loss. It is not clear, however, whether task switching was affected, since performance on two tasks that involve task switching were affected differently: Choice Reaction Time performance was impaired but Dual task performance was not.

The fact that C participants showed practice-related improvement on most executive control tasks on Day 2 raises a question of interpretation. There is a large literature demonstrating that performance on a wide array of learning tasks may improve after overnight sleep, presumably because of the benefits of sleep (or one or more sleep stages) on the process of consolidating newly acquired skills or information [for review, see (36)]. These effects are seen for sensory and motor learning and for a variety of tasks that involve explicit learning [e.g., (37, 38)]. It is unclear whether the practice effects observed in this study were related to changes in sensory processing, motor responses or strategic approaches to these tasks, but, in principle, any of these could reflect a form of incidental learning during the first experience of the tasks. If this learning were subject to sleep-related consolidation, relatively poorer performance by SR participants on Day 2 might reflect both direct effects of sleep loss on performance and indirect effects mediated by a lack of sufficient consolidation of skills or strategies acquired on Day 1. However, it is also possible that retesting participants later on Day 1 would have yielded similar improvements; i.e., that the improvement was not sleep-related. Different experimental approaches would be needed to evaluate these possibilities.

## Strengths and limitations

Among the strengths of this study are the use of one night of habituation to a novel environment to reduce any “first-night” effects on sleep quality (39–41), and testing baseline performance only after a second night of sleeping in the laboratory. We also used sleep diaries to ensure that participants did not experience unusual sleep timing or durations immediately before entering the study. Finally, the use of the DalCAB allowed assessment of a variety of cognitive mechanisms in a single study using an integrated testing approach (i.e., consistent stimuli, tasks purported to measure specific components of attention and all tests administered in a single session).

One limitation of this study is that, in the absence of polysomnography, we can only refer to time in bed (TIB) as an indication of sleep duration, rather than to actual sleep durations, nor do we have information on stages of sleep obtained in each condition. It is unlikely that participants slept a full 9 h when allowed to do so, but this duration was intended to allow them a typical, full night of sleep (reported as 7–9 h by participants). Control participants also reported sleeping typically during the nights with 9 h TIB. SR participants were accompanied by a researcher throughout their wake period, so they could not have slept more than 3 h on the SR night (04:00–07:00). The sleep manipulation was successful in inducing increased subjective sleepiness and fatigue (as measured by the SSS and POMS) in the SR but not the C group.

Because the night of sleep restriction followed immediately after the first test day, we cannot discriminate effects of sleep loss on next-day performance from effects of a reduced opportunity for sleep-related consolidation during the SR night. Although the tasks involved did not explicitly involve learning, improvement among C participants on Day 2 raises the possibility that some form of memory consolidation or skill improvement occurred, but there is no information as to whether it occurred during Day 1 testing, during subsequent waking or during the following night. Studies of nap effects on memory consolidation indicate that even short naps can provide an opportunity for memory consolidation that can affect next-day performance (42–44). Since we do not know whether improvement on Day 2 in controls was related to sleep, we also cannot speculate whether 3 h of sleep would provide an adequate consolidation opportunity.

Finally, the conclusions about effects of sleep loss on attention in this study are applicable only to the current sleep restriction paradigm (i.e., a single night with 3 h TIB). Future studies would have to evaluate whether protocols involving more extreme or repeated episodes of sleep loss would have different effects on the processes measured using these tests. In addition, there were baseline differences between groups in performance on a few tasks (feature visual search, Go/No-Go, item memory). It is unknown why these otherwise well-matched groups differed in this way. These baseline differences did not affect achieving the goal of the study, which was to examine how performance changed from baseline after sleep restriction or adequate sleep.

## Conclusions

A single night of sleep restriction to 3 h TIB increased daytime sleepiness and fatigue the next day. Associated with these changes were impairments in performance on tasks assessing vigilance and some executive processes related to visual attention (i.e., filtering, inhibition, working memory) but not others (task switching); no effects were observed on spatial orienting processes related to attention. These results imply that the relatively common experience of reduced sleep for a single night can have significant effects on several mechanisms related to attention, which can in turn affect many aspects of cognition. A secondary implication is that relatively modest, unintended sleep loss in study participants could affect performance on cognitive tasks, which may therefore not accurately reflect cognitive capacity that would be shown under sleep satiation conditions. This consideration may be especially relevant when testing populations with a variety of clinical conditions that are commonly associated with sleep disruption.

## Author contributions

JC: Study design, data collection, data analysis, and manuscript writing; SJ: Data analysis, manuscript writing, mentorship, and assistance; GE: Study design, data analysis, manuscript writing, and overall supervision; BR: Study design, data collection, data analysis, manuscript writing, and overall supervision.

### Conflict of interest statement

The authors declare that the research was conducted in the absence of any commercial or financial relationships that could be construed as a potential conflict of interest.

## Abbreviations

ANT: Attention Network Test
C: Control (group)
CRT: Choice reaction time
DalCAB: Dalhousie Computerized Attention Battery
GNG: Go/no-go
MEQ: Morningness-Eveningness Questionnaire
OSO: Overnight sleep opportunity
POMS: Profile of Mood States
PVT: Psychomotor Vigilance Task
RT: Reaction time
SR: Sleep restriction (group)
SRT: Simple reaction time
SSS: Stanford Sleepiness Scale
TIB: Time in bed.
