# Supplementary material for: Acute Sleep Restriction Has Differential Effects on Components of Attention
Source: Front Psychiatry. 2018 Oct 30;9:499. doi: 10.3389/fpsyt.2018.00499 (PMC6218409; doi:10.3389/fpsyt.2018.00499)
Supplement: Supplementary file 1 [file Data_Sheet_1.PDF]

Supplementary Material

Acute Sleep Restriction has Differential Effects on Components of Attention

Jasmyn E. A. Cunningham[1,3], Stephanie A. H. Jones[2], Gail A. Eskes[3,4], Benjamin Rusak*[3,4,5]

*** Correspondence:** Benjamin Rusak: rusak@dal.ca

1 Supplementary Figures and Tables.

Statistically significant differences are highlighted in bold text.

Table S1. Results of mixed design ANOVA analyses of Profile of Mood States (POMS) and Stanford Sleepiness Scale (SSS) data for Control and Sleep Restriction participants on mornings 1 and 2.

Scale/Item	Control		Sleep Restriction		Main Effects	Interactions
	Morning 1 Mean (SD)	Morning 2 Mean (SD)	Morning 1 Mean (SD)	Morning 2 Mean (SD)		
POMS Fatigue	3.4 (3.3)	3.4 (3.5)	4.3 (3.1)	9.0 (4.9)	**Group: $F_{(1, 36)}$=9.1, p=4.6x10^{-3}**<br>**Time: $F_{(1, 36)}$=14.9, p=4.5x10^{-4}**	**Group x Time: $F_{(1, 36)}$=13.4, p=7.9x10^{-4}**
POMS Vigor	-8.0 (6.1)	-6.0 (8.9)	-6.3 (4.2)	-3.9 (4.9)	Group: $F_{(1, 36)}$=1.2, p=2.9x10^{-1}<br>**Time: $F_{(1, 36)}$=5.4, p=2.6x10^{-2}**	Group x Time: $F_{(1, 36)}$=0.04, p=8.4x10^{-1}
POMS Depression	0.3 (0.7)	0.4 (0.9)	0.9 (1.7)	1.5 (2.4)	Group: $F_{(1, 36)}$=3.6, p=6.7x10^{-2}<br>Time: $F_{(1, 36)}$=1.2, p=2.8x10^{-1}	Group x Time: $F_{(1, 36)}$=0.5, p=4.9x10^{-1}
POMS Confusion	5.1 (3.2)	4.7 (3.6)	5.0 (1.8)	6.8 (3.7)	Group: $F_{(1, 36)}$=1.1, p=3.0x10^{-1}<br>Time: $F_{(1, 36)}$=2.5, p=1.2x10^{-1}	**Group x Time: $F_{(1, 36)}$=5.9, p=2.0x10^{-2}**
POMS Anger	0.2 (0.5)	0.5 (0.7)	0.7 (1.2)	1.0 (1.4)	Group: $F_{(1, 36)}$=2.6, p=1.1x10^{-1}<br>Time: $F_{(1, 36)}$=2.0, p=1.7x10^{-1}	Group x Time: $F_{(1, 36)}$=2.9x10^{-3}, p=9.6x10^{-1}
POMS Tension	2.5 (1.3)	3.6 (2.0)	4.1 (2.7)	5.1 (3.1)	**Group: $F_{(1, 36)}$=4.6, p=3.8x10^{-2}**<br>**Time: $F_{(1, 36)}$=8.3, p=6.5x10^{-3}**	Group x Time: $F_{(1, 36)}$=6.0x10^{-3}, p=9.4x10^{-1}
SSS	2.9 (1.2)	3.0 (1.2)	3.1 (0.9)	4.5 (1.4)	**Group: $F_{(1, 37)}$=6.4, p=1.6x10^{-2}**<br>**Time: $F_{(1, 37)}$=14.8, p=4.5x10^{-4}**	**Group x Time: $F_{(1, 37)}$=12.2, p=1.3x10^{-3}**

Table S2. Results of Tukey's Honestly Significant Differences (HSD) tests with 95% confidence intervals, for Profile of Mood States (POMS) and Stanford Sleepiness Scale (SSS) data.

	Comparison Group 1	Comparison Group 2	Difference	95% CI (lower, upper)	Adjusted p-value
Fatigue					
	**SR Morn. 2**	**C Morn. 2**	**5.5**	**2.3, 8.7**	**$1.5x10^{-4}$**
	C Morn. 1	C Morn. 2	$8.88x10^{-16}$	-3.3, 3.3	1.0
	**SR Morn. 1**	**SR Morn. 2**	**-4.7**	**-7.8, -1.6**	**$1.0x10^{-3}$**
	SR. Morn. 1	C Morn. 1	0.8	-2.4, 4.0	0.9
Vigor					
	SR Morn. 2	C Morn. 2	2.1	-3.2, 7.4	0.7
	C Morn. 1	C Morn. 2	-2.0	-7.4, 3.4	0.8
	SR Morn. 1	SR Morn. 2	-2.4	-7.5, 2.7	0.6
	SR. Morn. 1	C Morn. 1	1.7	-3.6, 7.0	0.8
Depression					
	SR Morn. 2	C Morn. 2	1.0	-0.4, 2.4	0.2
	C Morn. 1	C Morn. 2	-0.1	-1.5, 1.3	1.0
	SR Morn. 1	SR Morn. 2	-0.6	-1.9, 0.8	0.7
	SR. Morn. 1	C Morn. 1	0.6	-0.8, 1.9	0.7
Confusion					
	SR Morn. 2	C Morn. 2	2.1	-0.6, 4.8	0.2
	C Morn. 1	C Morn. 2	0.4	-2.3, 3.2	1.0
	SR Morn. 1	SR Morn. 2	-1.8	-4.4, 0.8	0.3
	SR. Morn. 1	C Morn. 1	-0.2	-2.8, 2.5	1.0
Anger					
	SR Morn. 2	C Morn. 2	0.5	-0.4, 1.3	0.6
	C Morn. 1	C Morn. 2	-0.3	-1.2, 0.6	0.9
	SR Morn. 1	SR Morn. 2	-0.3	-1.2, 0.6	0.8
	SR. Morn. 1	C Morn. 1	0.4	-0.5, 1.3	0.6
Tension					
	SR Morn. 2	C Morn. 2	1.5	-0.6, 3.6	0.2
	C Morn. 1	C Morn. 2	-1.1	-3.2, 1.1	0.6
	SR Morn. 1	SR Morn. 2	-1.0	-3.0, 1.0	0.6
	SR. Morn. 1	C Morn. 1	1.6	-0.5, 3.6	0.2
SSS					
	**SR Morn. 2**	**C Morn. 2**	**1.5**	**0.5, 2.5**	**$9.8x10^{-4}$**
	C Morn. 1	C Morn. 2	0.05	-1.0, 1.1	1.0
	**SR Morn. 1**	**SR Morn. 2**	**1.4**	**0.4, 2.4**	**$2.0x10^{-3}$**
	SR. Morn. 1	C Morn. 1	0.2	-0.8, 1.1	1.0

Table S3. Results of mixed design ANOVA analyses of DalCAB RT data for Control and Sleep Restriction participants on mornings 1 and 2.

Task	Control		Sleep Restriction		Main Effects	Interactions
	Morning 1 Mean (SD)	Morning 2 Mean (SD)	Morning 1 Mean (SD)	Morning 2 Mean (SD)		
Simple RT	259.6 (77.4)	264.3 (75.7)	260.7 (68.8)	283.1 (79.6)	Group: $F_{(1, 37)}=0.98$, p=0.33<br>**Time: $F_{(1, 37)}=7.53$, p=9.32x10^{-3}**<br>**RSI[*]: $F_{(2, 74)}=94.61$, p=4.07x10^{-21}**	Group x Time: $F_{(1, 37)}=3.16$, p=0.08<br>Group x RSI: $F_{(2, 74)}=0.36$, p=0.70<br>**RSI x Time: $F_{(2, 74)}=4.74$, p=1.16x10^{-2}**<br>Group x RSI x Time: $F_{(2, 74)}=0.58$, p=0.56
Choice RT	426.2 (135.8)	385.8 (113.4)	425.6 (125.2)	413.3 (120.9)	Group: $F_{(1, 37)}=0.81$, p=0.37<br>**Time: $F_{(1, 37)}=20.96$, p=5.15x10^{-5}**<br>**Response Switch: $F_{(1, 37)}=115.03$, p=6.57x10^{-13}**	**Group x Time: $F_{(1, 37)}=5.99$, p=1.92x10^{-2}**<br>Group x Response Switch: $F_{(1, 37)}=0.45$, p=0.51<br>Response Switch x Time: $F_{(1, 37)}=0.69$, p=0.41<br>Group x Response Switch x Time: $F_{(1, 37)}=0.33$, p=0.57
Feature Visual Search	676.5 (304.0)	597.0 (163.5)	651.1 (172.1)	619.8 (166.2)	Group: $F_{(1, 37)}=4.08$x10^{-3}, p=0.95<br>**Time: $F_{(1, 37)}=35.61$, p=6.95x10^{-7}**<br>**Set Size: $F_{(2, 74)}=3.16$, p=4.84x10^{-2}**	**Group x Time: $F_{(1, 37)}=6.91$, p=1.24x10^{-2}**<br>Group x Set Size: $F_{(2, 74)}=7.50$x10^{-3}, p=0.99<br>Set Size x Time: $F_{(2, 74)}=2.71$, p=0.07<br>Group x Set Size x Time: $F_{(2, 74)}=0.02$, p=0.98
Conjunction Visual Search	1428.7 (797.1)	1230.2 (643.3)	1455.8 (927.4)	1333.4 (828.6)	Group: $F_{(1, 37)}=1.00$, p=0.32<br>**Time: $F_{(1, 37)}=67.70$, p=7.02x10^{-10}**<br>**Set Size:**	Group x Time: $F_{(1, 37)}=3.71$, p=6.18x10^{-2}<br>Group x Set Size:

					**$F_{(2, 74)}$=255.07, p=6.31x10^{-34}**	$F_{(2, 74)}$=2.07, p=0.13<br>**Set Size x Time:**<br>**$F_{(2, 74)}$=7.64, p=9.64x10^{-4}**<br>Group x Set Size x Time:<br>$F_{(2, 74)}$=0.03, p=0.97
Dual Task - Primary	512.0 (218.1)	470.3 (193.3)	535.8 (226.8)	487.7 (198.8)	Group: $F_{(1, 37)}$=0.19, p=0.67<br>**Time: $F_{(1, 37)}$=27.95, p=5.80x10^{-6}**<br>**Response Switch:**<br>**$F_{(1, 37)}$=313.16, p=1.20x10^{-19}**	Group x Time:<br>$F_{(1, 37)}$=0.72, p=0.40<br>Group x Response Switch:<br>$F_{(1, 37)}$=3.52x10^{-2}, p=0.85<br>Response Switch x Time:<br>$F_{(1, 37)}$=2.33, p=0.13<br>Group x Response Switch x Time: $F_{(1, 37)}$=0.12, p=0.73
Dual Task - Secondary	1627.2 (777.7)	1477.6 (633.5)	1679.5 (887.6)	1554.6 (931.3)	Group: $F_{(1, 37)}$=0.80, p=0.38<br>**Time: $F_{(1, 37)}$=7.23, p=0.01**	Group x Time:<br>$F_{(1, 37)}$=0.05, p=0.82
Flanker Task	461.2 (128.6)	437.1 (131.7)	463.0 (118.0)	466.1 (128.8)	Group: $F_{(1, 37)}$=0.54, p=0.47<br>Time: $F_{(1, 37)}$=2.73, p=0.11<br>**Congruency:**<br>**$F_{(1, 37)}$=28.28, p=5.26x10^{-6}**	**Group x Time:**<br>**$F_{(1, 37)}$=6.49, p=1.52x10^{-2}**<br>Group x Congruency:<br>$F_{(1, 37)}$=0.11, p=0.75<br>Congruency x Time:<br>$F_{(1, 37)}$=0.02, p=0.88<br>**Group x Congruency x Time:**<br>**$F_{(1, 37)}$=4.84, p=0.03**
Go/No-Go	326.8 (83.3)	316.6 (83.1)	340.1 (81.6)	355.3 (95.1)	**Group: $F_{(1, 37)}$=6.84, p=0.01**<br>Time: $F_{(1, 37)}$=2.94, p=0.09<br>**Go Frequency:**<br>**$F_{(1, 37)}$=181.64, p=7.58x10^{-16}**	**Group x Time:**<br>**$F_{(1, 37)}$=23.25, p=2.44x10^{-5}**<br>Group x Go Frequency:<br>$F_{(1, 37)}$=1.36, p=0.25<br>Go Frequency x Time:<br>$F_{(1, 37)}$=3.54, p=0.07<br>Group x Go Frequency x Time: $F_{(1, 37)}$=3.97, p=0.05
Item Memory	1005.5 (439.2)	882.4 (377.7)	876.3 (374.6)	829.1 (348.1)	Group: $F_{(1, 37)}$=3.43, p=0.07<br>**Time: $F_{(1, 37)}$=17.61, p=1.63x10^{-4}**<br>**Set Size:**	**Group x Time:**<br>**$F_{(1, 37)}$=4.60, p=3.85x10^{-2}**<br>Group x Set Size:

					**$F_{(2, 74)}$=158.35, p=1.83x10^{-27}**	$F_{(2, 74)}$=1.61, p=0.21<br>Set Size x Time:<br>$F_{(2, 74)}$=0.26, p=0.78<br>Group x Set Size x Time:<br>$F_{(2, 74)}$=0.15, p=0.86
Location Memory	840.6 (340.0)	787.3 (307.0)	833.0 (337.2)	810.0 (338.4)	Group: $F_{(1, 37)}$=0.02, p=0.88<br>Time: $F_{(1, 37)}$=2.78, p=0.10<br>**Set Size:**<br>**$F_{(2, 74)}$=38.68, p=3.18x10^{-12}**	Group x Time:<br>$F_{(1, 37)}$=1.05, p=0.31<br>Group x Set Size:<br>$F_{(2, 74)}$=0.95, p=0.39<br>Set Size x Time:<br>$F_{(2, 74)}$=1.12, p=0.33<br>Group x Set Size x Time:<br>$F_{(2, 74)}$=1.13, p=0.33

*Response Stimulus Interval.

Table S4. Results of Tukey's Honestly Significant Differences (HSD) tests with 95% confidence intervals for DalCAB RT data.

	Comparison Group 1	Comparison Group 2	Difference	95% CI (lower, upper)	Adjusted p-value
Simple RT					
	C Morn. 2	C Morn. 1	4.8	-3.5, 13.0	0.45
	SR Morn. 1	C Morn. 1	1.1	-7.0, 9.2	0.99
	**SR Morn. 2**	**C Morn. 2**	**18.8**	**10.7, 26.9**	**5.89×10^{-8}**
	**SR. Morn. 2**	**SR. Morn. 1**	**22.5**	**14.5, 30.5**	**4.20×10^{-8}**
Choice RT					
	**C Morn. 2**	**C Morn. 1**	**-40.3**	**-50.0, -30.7**	**0.00**
	SR Morn. 1	C Morn. 1	-0.5	-10.0, 9.0	1.00
	**SR Morn. 2**	**C Morn. 2**	**27.5**	**17.9, 37.0**	**0.00**
	**SR. Morn. 2**	**SR. Morn. 1**	**-12.3**	**-21.7, -2.9**	**0.00**
Feature Visual Search	**C Morn. 2**	**C Morn. 1**	**-79.5**	**-96.7, -62.3**	**2.57×10^{-11}**
	**SR Morn. 1**	**C Morn. 1**	**-25.4**	**-42.4, -8.4**	**7.07×10^{-4}**
	**SR Morn. 2**	**C Morn. 2**	**22.8**	**5.7, 39.8**	**3.35×10^{-3}**
	**SR. Morn. 2**	**SR. Morn. 1**	**-31.4**	**-48.2, -14.6**	**9.58×10^{-6}**
Conjunction Visual Search	**C Morn. 2**	**C Morn. 1**	**-198.6**	**-264.8, -132.4**	**2.52×10^{-11}**
	SR Morn. 1	C Morn. 1	27.1	-38.3, 92.5	0.71
	**SR Morn. 2**	**C Morn. 2**	**103.3**	**37.9, 168.7**	**2.94×10^{-4}**
	**SR. Morn. 2**	**SR. Morn. 1**	**-122.4**	**-187.0, -57.8**	**6.83×10^{-6}**
Dual Task - Primary	**C Morn. 2**	**C Morn. 1**	**-41.7**	**-54.4, -28.9**	**8.75×10^{-9}**
	**SR Morn. 1**	**C Morn. 1**	**23.9**	**11.3, 36.4**	**5.96×10^{-6}**
	**SR Morn. 2**	**C Morn. 2**	**17.4**	**4.8, 30.0**	**2.24×10^{-3}**
	**SR. Morn. 2**	**SR. Morn. 1**	**-48.1**	**-60.5, -35.8**	**8.75×10^{-9}**
Dual Task - Secondary	C Morn. 2	C Morn. 1	-149.6	-313.4, 14.3	8.80×10^{-2}
	SR Morn. 1	C Morn. 1	52.4	-113.7, 218.4	0.85
	SR Morn. 2	C Morn. 2	77.0	-84.0, 237.9	0.61
	SR. Morn. 2	SR. Morn. 1	-125.0	-288.1, 38.2	0.20
Flanker Task					
	**C Morn. 2**	**C Morn. 1**	**-24.1**	**-35.0, -13.2**	**8.26×10^{-8}**
	SR Morn. 1	C Morn. 1	1.8	-8.9, 12.6	0.97
	**SR Morn. 2**	**C Morn. 2**	**29.0**	**18.2, 39.8**	**3.84×10^{-11}**
	SR. Morn. 2	SR. Morn. 1	3.1	-7.6, 13.7	0.88
Go/No-Go					
	**C Morn. 2**	**C Morn. 1**	**-10.2**	**-17.4, -3.0**	**1.50×10^{-3}**
	**SR Morn. 1**	**C Morn. 1**	**13.4**	**6.3, 20.4**	**7.82×10^{-6}**
	**SR Morn. 2**	**C Morn. 2**	**38.7**	**31.6, 45.8**	**2.01×10^{-11}**
	**SR. Morn. 2**	**SR. Morn. 1**	**15.2**	**8.2, 22.2**	**1.59×10^{-7}**

Item Memory	**C Morn. 2**	**C Morn. 1**	**-123.1**	**-168.1, -78.1**	**$2.09x10^{-8}$**
	**SR Morn. 1**	**C Morn. 1**	**-129.2**	**-174.1, -84.4**	**$2.09x10^{-8}$**
	**SR Morn. 2**	**C Morn. 2**	**-53.3**	**-98.0, -8.6**	**$1.18x10^{-2}$**
	**SR. Morn. 2**	**SR. Morn. 1**	**-47.2**	**-91.8, -2.7**	**0.03**
Location Memory	**C Morn. 2**	**C Morn. 1**	**-53.3**	**-92.6, -14.0**	**$2.82x10^{-3}$**
	SR Morn. 1	C Morn. 1	-7.7	-46.9, 31.6	0.96
	SR Morn. 2	C Morn. 2	22.6	-16.7, 62.0	0.45
	SR. Morn. 2	SR. Morn. 1	-23.0	-62.2, 16.3	0.43
